# Minimal genetic change in *Vibrio cholerae* in Mozambique over time: Multilocus variable number tandem repeat analysis and whole genome sequencing

**DOI:** 10.1371/journal.pntd.0005671

**Published:** 2017-06-16

**Authors:** Marcelino Garrine, Inácio Mandomando, Delfino Vubil, Tacilta Nhampossa, Sozinho Acacio, Shan Li, Joseph N. Paulson, Mathieu Almeida, Daryl Domman, Nicholas R. Thomson, Pedro Alonso, Oscar Colin Stine

**Affiliations:** 1 Centro de Investigação em Saúde de Manhiça (CISM), Maputo, Mozambique; 2 Instituto Nacional de Saúde (INS), Ministério da Saúde, Maputo, Mozambique; 3 Department of Epidemiology and Public Health, University of Maryland Baltimore, Baltimore, Maryland, United States of America; 4 Institute for Applied Computer Sciences, University of Maryland, College Park, Maryland, United States of America; 5 Department of Biostatistics, Dana-Farber Cancer Institute, Boston, Massachusetts, United States of America; 6 Infection Genomics, Wellcome Trust Sanger Instititue, Hinxton, England, United Kingdom; 7 ISGlobal Barcelona Ctr. Int. Health Res. (CRESIB), Hospital Clínic / Universitat de Barcelona, Barcelona, Spain; University of Oxford, UNITED KINGDOM

## Abstract

Although cholera is a major public health concern in Mozambique, its transmission patterns remain unknown. We surveyed the genetic relatedness of 75 *Vibrio cholerae* isolates from patients at Manhiça District Hospital between 2002–2012 and 3 isolates from river using multilocus variable-number tandem-repeat analysis (MLVA) and whole genome sequencing (WGS). MLVA revealed 22 genotypes in two clonal complexes and four unrelated genotypes. WGS revealed i) the presence of recombination, ii) 67 isolates descended monophyletically from a single source connected to Wave 3 of the Seventh Pandemic, and iii) four clinical isolates lacking the cholera toxin gene. This Wave 3 strain persisted for at least eight years in either an environmental reservoir or circulating within the human population. Our data raises important questions related to where these isolates persist and how identical isolates can be collected years apart despite our understanding of high change rate of MLVA loci and the *V*. *cholerae* molecular clock.

## Author summary

Cholera is a deadly disease caused by the bacterium *Vibrio cholerae*. The ancestral home of cholera is around the Bay of Bengal, but recently cholera has moved to Africa. In Africa, cholera occurs in sporadic outbreaks. In order prevent cases of cholera, we want to understand the transmission of cholera in Africa, does it stay in one place or does it move around. To gain insight into these questions, we have examined the DNA of the bacteria. The DNA provides a identity for each isolate and we can infer how the isolates are related to each other based on the number and type of DNA changes. In our study, we examined the DNA of cholera isolates from southern Mozambique. We were surprised how similar all the Mozambique isolates were even though that were collected up to eight years apart. Based on previous work, we would have expected much more change in the DNA. Our data raises several important questions that relate to where these cholera isolates persist, possibly in local refuges, and how seemingly identical isolates can be collected years apart despite our understanding of the high rate of change of the molecular clock.

## Introduction

Cholera remains a public health concern in developing countries with an estimated burden of 1.2–4.3 million cases and 28,000–142,000 deaths per year, worldwide [1]. South Asia and sub-Saharan Africa account with the majority of cases and deaths. Between 01 January and 03 June 2013, a total of 25,762 cholera cases and 490 deaths were reported from 18 African countries. Mozambique accounted for 7% (1,861/25,762) of cases and 4% (19/490) of deaths, being the third most affected country after the Democratic Republic of the Congo and Angola [2]. The most recent cholera outbreak in Mozambique started in December 2014 in Nampula [3], where there were 8,835 cases with case fatality rate of 0.7% (65 deaths) in 5 northern and central provinces in five months [4,5]. The most recent cholera outbreak in the south was reported in 2011 [6]. The peak of a cholera epidemic is often preceded by increasing prevalence of the pathogenic strains in the environment [7] where *V*. *cholerae* are harbored in aquatic reservoirs during extended periods between outbreaks [8].

*Vibrio cholerae* O1 is associated with most epidemic and pandemic outbreaks [9]. Whole genome sequence (WGS) analysis of *V*. *cholerae* isolates from around the world [9–12] demonstrated that the current (Seventh) Pandemic is monophyletic and originated from a single source with a clonal expansion of the lineage, with a strong temporal signature [13]. The seventh pandemic has been divided into 3 waves beginning in 1952, 1981 and 1988, respectively [13]. Each wave appears to be a selective sweep, as was the second wave of *V*. *cholerae* that swept across Haiti after the initial introduction [14]. In Africa, isolates from all three waves have been identified [13] with wave 3 isolates forming two distinct clades in Kenya between 2005 to 2010 [15].

The epidemiology and transmission patterns of outbreaks of *V*. *cholerae* have been explored using both multilocus variable number tandem repeat analysis (MLVA) and WGS [13,16]. In rural Bangladesh, MLVA revealed that multiple genetic lineages of *V*. *cholerae* occur naturally in the environment with geographic and seasonal genetic variation and identical genotypes can be found in the environment and humans [12]. In Kenya, MLVA demonstrated that several distinct genetic lineages emerged simultaneously during outbreaks in a single cholera season, linked to local environmental reservoirs [17]. WGS revealed all lineages were part of the Seventh Pandemic expansion [15]. In central and western Africa, MLVA revealed distinct clusters of isolates from different countries: Democratic Republic of the Congo, Zambia, Togo, and Guinea [18]. A second study in Guinea demonstrated spread and differentiation of *V*. *cholerae* during an outbreak [19].

In Mozambique, previous analysis of *V*. *cholerae* O1 showed these isolates had the typical traits of the El Tor biotype overall except that they carried a tandem array of classical CTX prophage [20] located on the small chromosome [21,22]. However, there are no data available on the genetic relatedness of *V*. *cholerae* circulating in Manhiça in southern Mozambique. Here, we characterized clinical and environmental *V*. *cholerae* isolates from Mozambique using MLVA and WGS to determine the genetic relatedness of strains isolated from patients with diarrhea in Manhiça District Hospital.

## Methodology

### Site description

Manhiça District Hospital (MDH) is a 110 bed referral health facility for Manhiça District, a rural area of Maputo Province in southern Mozambique. The characteristics of the area have been described in detail elsewhere [23,24]. Briefly, the climate is subtropical with two distinct seasons: a warm, rainy season between November and April, and a cool and dry season during the rest of the year. Manhiça has 160,000 inhabitants, who are mostly subsistence farmers or workers in two large sugar- and fruit-processing factories. The Manhiça Health Research Centre (Centro de Investigação em Saúde da Manhiça[CISM]) is adjacent to the MDH and has been conducting continuous demographic surveillance for vital events and migrations since 1996 [23], currently covering 165,000 individuals.

### Ethics statement

The strain collection of *V*. *cholerae* described in the study was isolated from cholera surveillance and other studies conducted in the Manhiça community by CISM approved by the National BioEthic Committee (CNBS). Any and all patient data were anonymized/de-identified. The IRB at University of Maryland School of Medicine approved the use of anonymized strains.

### Bacterial isolates

A total of 75 *V*. *cholerae* isolates were collected from MDH, between 2002 and 2012. The strains were isolated from stool of patients admitted to the MDH with suspicion of cholera, presenting with watery diarrhea. In addition, three isolates collected from the Incomati River were included. *V*. *cholerae* isolates were identified by standard biochemical tests; and confirmed by API-20E biochemical test strips (bioMérieux SA, Marcy-l'Etoile, France). Serotypes were determined using commercially available poly- and mono-clonal slide agglutination antisera (Mast Group Ltd., Merseyside, UK) according to the manufacturer’s instructions. All the isolates were stored at -80°C in tryptone soya broth (TSB) with 15% glycerol, and retrieved at the time for molecular characterization.

### Preparation of bacterial DNA

A pure culture of *V*. *cholerae* was plated in Thiosulfate Citrate Bile Sucrose (TCBS) agar and incubated overnight at 37°C. DNA was extracted using the Qiagen QIAamp DNA Mini Kit (Hilden, Germany). The DNA template was sent to the University of Maryland Baltimore, Baltimore, Maryland, USA for molecular typing by MLVA and WGS.

### MLVA

DNA from each isolate was amplified by PCR using the conditions and primers previously described for 5 loci containing variable length tandem repeats [11]. The amplified products were separated and detected using a model 3730xl Automatic Sequencer (ABI) and their sizes were determined using internal lane standards (Liz600; ABI, Foster City, CA) with the Gene Mapper v4.0 program (ABI). Genotypes were determined according to the published formulas to calculate the number of repeats from the length of each allele and identify the alleles at the 5 loci [11]. The 5 loci, in order, are VC0147, VC0436–7 (intergenic), VC1650, VCA0171, and VCA0283; thus, the genotype 9,4,6,19,11 indicates that the isolate has alleles of 9, 4, 6, 19, and 11 repeats at the 5 loci, respectively. Relatedness of the strains was assessed by eBURSTv3 (http://eburst.mlst.net), in which genetically related genotypes were defined as those possessing at least 4 identical alleles of the 5 loci. An alternative analysis was performed using Network 2.x (http://www.fluxus-engineering.com/sharefaq.htm).

### Whole genome sequencing

The DNA concentration was quantified by NanoDrop 2000 Spectrophotometer (Thermofisher Scientific, Waltham, MA, USA) and only specimens with sufficient concentration (n = 71) were submitted to WGS. DNA was prepared for Illumina sequencing using the KAPA High Throughput Library Preparation Kit (KapaBiosystems, Wilmington, MA). DNA was fragmented with the Covaris E210. Libraries were prepared using a modified version of manufacturer’s with-bead protocol (KapaBiosystems, Wilmington, MA). The libraries were enriched and barcoded by ten cycles of PCR amplification step with primers containing an index sequence seven nucleotides in length. The libraries were sequenced on a 100 bp paired-end run on an Illumina HiSeq2500 (Illumina, San Diego, CA).

The quality of the 101-base paired-end reads was confirmed using Fastqc (https://www.bioinformatics.babraham.ac.uk/projects/fastqc/). Kmergenie (http://kmergenie.bx.psu.edu/) was used for choice of the best peak and the assembly was performed using the SPAdes software [25]. CSI Phylogeny 1.0a (http://cge.cbs.dtu.dk/services/CSIPhylogeny/) was used to generate a tree of genetic relatedness based on high quality nucleotide variants and then compared to *V*. *cholerae* O1 El Tor reference strain N16961 (NCBI accession numbers AE003852 and AE003853). Splitstree [26] was used to determine networks. Previous work in our lab demonstrated that this pipeline produces identical results to SMALT [27].

A high resolution SNP based phylogeny for the 67 7^th^ pandemic strains was placed in context of a globally representative collection of 274 isolates (S1 Table) by mapping the reads to the *V*. *cholerae* 01 El Tor reference N16961 using SMALT (http://www.sanger.ac.uk/resources/software/smalt) as previously described [28]. Gubbins [29] was used to simultaneously remove regions of high SNP density and putative recombination sites in the alignment and infer the phylogenetic tree. The pre-seventh pandemic strain M66 (NCBI accession numbers CP001233 and CP001234) was used to root the phylogenetic tree.

To accurately place the four non-01 *V*. *cholerae* into a phylogenetic context we calculated a core genome alignment of 1093 genes (1,055,747 bp) using Roary [30] from a set of diverse *V*. *cholerae* genomes along with genomes of the closely related species *Vibrio metoecus* and *Vibrio parilis*. The resulting alignment was used to reconstruct the phylogenetic relationship using RAxML v. 7.8.6 [31] under the GTR model with 100 bootstrap replicates. The resulting phylogenetic trees were visualized using FigTree v.1.4.2 (http://tree.bio.ed.ac.uk/software/figtree/).

## Results

Among the 78 isolates (Table 1), 26 distinct genotypes were identified by MLVA using all five VNTR loci. The numbers of distinct alleles at loci VC0147, VC0437, VC1650, VCA0171, and VCA0283 were 10, 2, 4, 7 and 7, respectively.

**Table 1:** Vibrio cholerae isolates and their genetic characterization.

specimen ID	sample	collection date	P1	P2	P3	P4	P5	Total reads	# Contigs	Total length	N50	# predicted genes (> = 300 bp)
0005	stool	12-May-02	8	4	6	18	21	15822446	124	4042725	235749	3232
0010	river	14-May-02	8	4	6	18	21	15170986	153	4162168	235741	3345
0014	river	14-May-02	2	4	6	18	21	17042970	145	4041694	235689	3231
0015	stool	20-May-02	8	4	6	18	21	15170986	116	4040728	168110	3229
0016	stool	20-May-02	2	4	6	19	21	12570276	126	4041525	235753	3234
0017	stool	20-May-02	8	4	6	18	21	16953980	129	4039582	246625	3229
0018	stool	20-May-02	8	4	6	18	21	27277596	134	4039159	235697	3230
0019	stool	21-May-02	8	4	6	18	21	17097584	135	4037123	235733	3224
0034	stool	7-Jun-02	2	4	6	18	22	17456602	153	4045314	215343	3233
0035	stool	7-Jul-02	2	4	6	18	21	14346844	110	4043196	274420	3230
0074	stool	4-Mar-03	8	4	6	18	21	16015980	109	4042919	299283	3233
0079	stool	5-Mar-03	8	4	6	18	21	14829360	128	4048089	246625	3234
0008	river	14-May-05	8	4	6	17	21	15220440	118	4038347	235757	3229
0091	stool	15-Mar-03	8	4	6	18	21	16345390	117	4044727	235757	3233
091	stool	NA	5	4	6	16	22	15007662	134	4170716	198190	3357
100	stool	21-Mar-03	6	4	6	18	21	15306054	117	4047306	235773	3235
101	stool	21-Mar-03	8	4	6	18	21	17234372	130	4043196	235733	3232
1019828.5	stool	4-Jun-10	8	4	6	18	22	17051454	113	4044240	246637	3236
1019829.2	stool	4-Jun-10	2	4	6	18	22	11946036	157	4041334	235721	3233
1020229.6	stool	1-Jun-10	3	4	6	18	22	15571312	107	4042920	299307	3230
1020231.9	stool	1-Jun-10	8	4	6	18	22	14358586	188	4038634	197527	3230
1020234.0	stool	1-Jun-10	2	4	6	18	22	16248046	115	4044859	246637	3235
105	stool	23-Mar-03	9	4	6	18	24	14272564	152	4199705	235741	3379
1163068.5	stool	02-Mar-12	10	4	3	14	14	14358586	205	4028212	244702	3213
120	stool	7-Apr-03	2	4	6	18	21	14611668	119	4044669	198208	3233
121	stool	9-Apr-03	8	4	6	18	21	13629302	123	4042373	240831	3232
122	stool	11-Apr-03	2	4	6	19	21	16249350	136	4041110	235737	3229
134	stool	20-Apr-03	9	4	6	18	10	15496714	111	4202583	274384	3386
146	stool	2-May-03	9	4	6	18	23	14094190	111	4201741	246452	3387
147	stool	2-May-03	5	4	6	18	24	18769590	139	4205115	166699	3389
151	stool	5-May-03	8	4	6	18	21	13435326	134	4046368	165090	3235
152	stool	5-May-03	8	4	6	18	21	11486728	114	4038231	246645	3226
153	stool	5-May-03	8	4	6	18	21	19344048	131	4166028	246625	3348
154	stool	6-May-03	8	4	6	18	21	13787496	126	4040931	246629	3232
178	stool	15-May-03	9	4	6	18	24	13855938	136	4197109	215352	3381
179	stool	16-May-03	9	4	6	18	23	16884124	143	4203535	235745	3385
188	stool	17-May-03	9	4	6	18	24	16306802	138	4205908	166695	3389
189	stool	17-May-03	6	4	6	18	24	15775962	229	4198382	176073	3378
191	stool	19-May-03	6	4	6	18	23	17116600	117	4201351	246476	3385
196	stool	21-May-03	5	4	6	18	24	16031708	126	4204296	246464	3384
296	stool	NA	2	4	6	18	22	15853624	177	4040423	235697	3234
300205	stool	11-Jan-08	7	4	14	13	21	33740130	328	3951968	246629	3131
300043	stool	16-Jan-08	8	4	6	18	21	10140136	131	4046708	185889	3234
300055	stool	27-Jan-08	13	4	7	12	21	9175956	240	4159518	133584	3331
300208	stool	24-Jan-08	8	4	6	18	21	12186024	118	4047015	168139	3234
300209	stool	25-Jan-08	8	4	6	19	21	12428824	110	4044616	299303	3232
300215	stool	19-Feb-08	8	4	6	18	21	11271788	168	4073917	246641	3246
300506	stool	27-Apr-08	12	12	3	17	21	9274148	173	4040231	339296	3217
302015	stool	16-Feb-09	9	4	6	18	23	10980664	118	4214484	246476	3393
302029	stool	5-Mar-09	9	4	6	18	21	11199308	174	4229711	274301	3394
326	stool	NA	8	4	6	18	20	15654426	124	4165971	246637	3348
347	stool	NA	8	4	6	18	21	15286606	146	4051922	246633	3227
374	stool	NA	8	4	6	18	21	8013166	141	4043250	299283	3225
375	stool	NA	8	4	6	19	21	17973118	173	4041898	215270	3229
382	stool	NA	8	4	6	18	21	17226956	130	4046077	173229	3234
398	stool	NA	8	4	6	16	21	17282880	443	4102857	114507	3259
399	stool	NA	8	4	6	18	21	15570318	99	4038494	274422	3227
420	stool	NA	8	4	6	18	21	18048184	161	4040168	225617	3234
505	stool	NA	8	4	6	18	21	16442290	119	4047614	246645	3234
510	stool	NA	8	4	6	18	21	16619422	117	4041499	216033	3229
511	stool	NA	2	4	6	18	21	19004672	136	4043523	235737	3233
655630.3	stool	NA	8	4	6	18	21	14338294	178	4070175	246637	3247
655664.3	stool	NA	8	4	6	18	22	13650350	142	4175603	166703	3357
655665.0	stool	NA	2	4	6	18	21	17379870	113	4044785	246633	3235
710180.8	stool	NA	7	4	6	16	22	16589666	111	4201582	235757	3385
740115.4	stool	NA	8	4	6	18	22	16126366	117	4043435	298463	3233
769845.7	stool	NA	8	4	6	18	23	15919268	124	4204461	299303	3385
770067.4	stool	NA	6	4	6	18	24	9609488	133	4201831	235749	3385
770180.8	stool	NA	7	4	6	16	22	15532364	116	4198654	212820	3386
780298.0	stool	NA	6	4	6	18	23	14033966	136	4203224	167915	3386
S/Nida	stool	NA	5	4	6	18	24	15541740	140	4205161	208032	3388
0092	stool	15-Mar-03	5	4	6	18	20					
0006	stool	NA	8	4	6	18	21					
0020	stool	NA	8	4	6	18	21					
0093	stool	NA	2	4	6	18	21					
201	stool	NA	8	4	6	18	21					
271	stool	NA	2	4	6	18	22					
333	stool	NA	2	4	6	18	21					
Average								15375291	143	4094492	232090	3279

**Fig 1:**
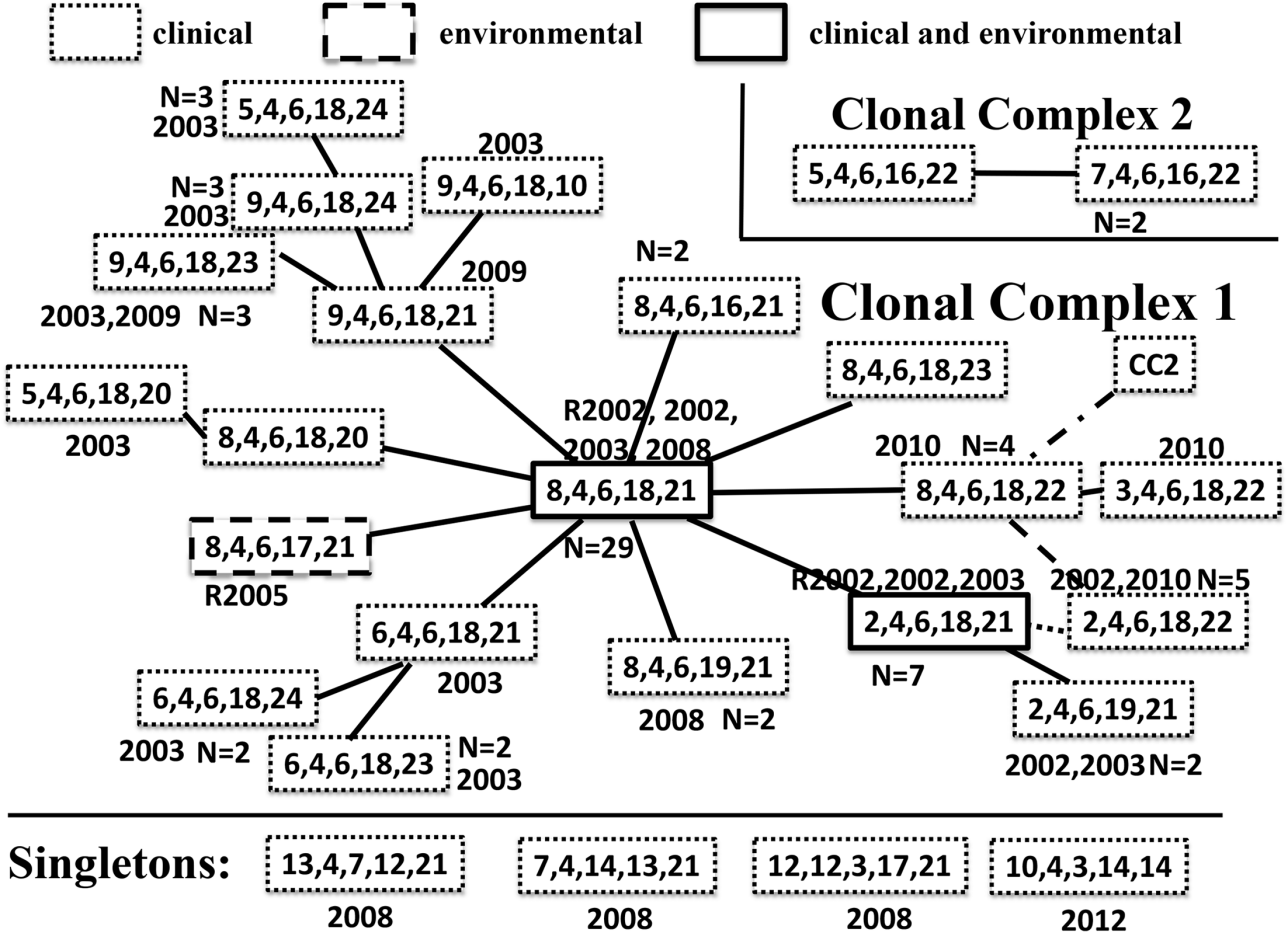
Genetic relatedness between *V*. *cholerae* genotypes by MLVA, Mozambique, 2002–2012. Each box with five numbers represents a genotype; each connecting line, an allelic change at a single locus. The solid boxes are around genotypes found in both the river and in patients, dotted boxes are for genotypes found only in patients and dashed boxes for genotypes found only in river isolates (R). The numbers outside the boxes indicate the year or years isolates with that genotype were found. There are twenty genotypes in CC1, two genotypes in CC2 and four singleton genotypes unrelated to any other genotypes using eBURST. An alternative analysis using Network revealed the following differences: i) a connection in eBURST, but not in Network, was represented by a dotted line (single locus change), ii) a connection in Network, but not in eBURST, by a dashed line (single locus change), iii) in Network, but not in eBURST, CC2 is connected to CC1, we have represented this by a dot and dashed line (representing changes at two loci), finally, iv) in Network, but not in eBURST, all three singleton genotypes are connected to the founder genotype regardless of whether the singleton genotype differs at three or four loci from the founder.

Two clonal complexes of genetically related genotypes (each complex comprising genotypes that differed by an allelic change at a single locus) and four singleton genotypes unrelated to any other (differed by ≥2 loci) were identified (Fig 1). The first clonal complex (CC1) consisted of twenty of the twenty-six genotypes and comprised 91% (71/78) of the isolates (including all 3 isolates from river water). In CC1 the most common genotype, identified as the “founder” genotype (defined by eBURST as the genotype that differed from the largest number of other genotypes at a single locus), comprised 41% (29/71) of the isolates. In this complex, the founder genotype radiated into 9 other genotypes, and 5 of those differentiated further. Two of the three river water isolates shared a common genotype with the clinical isolates, one of which was of the founder genotype. Interestingly we found isolates with identical MLVA genotypes up to 8 years apart. In general, isolates from the same year were genetically related, all four genotypes found in 2002 were single locus variants, as were the two genotypes in 2008, two in 2009, and three genotypes from 2010. In contrast to similarity within a year, in months (May 2002, March 2003, April 2003, May 2003, June 2010) where multiple cases presented with *V*. *cholerae*, we found that isolates belonged to more than one genotype.

The other *V*. *cholerae* isolates consisted of a second clonal complex CC2 and four singletons (Fig 1). CC2 had two genotypes and comprised only 4% (3/78) of the analyzed isolates. The four singleton genotypes contained one isolate each corresponding to 5% (4/78) of the isolates. Three of them were isolated in 2008 and the fourth in 2012.

We successful sequenced the whole genomes of 71 *V*. *cholerae* O1 isolates (Table 1). The average number of high quality reads was 15,375,291 which upon assembly produced an average of 143 contigs (range: 99 to 443) or scaffolds with an average depth of 374. The assembled genome was 4.09 Mb (3.95 to 4.23 Mb) in length and had an average N50 of 232,090 bp (144,507 to 339,296 bp).

**Fig 2:**
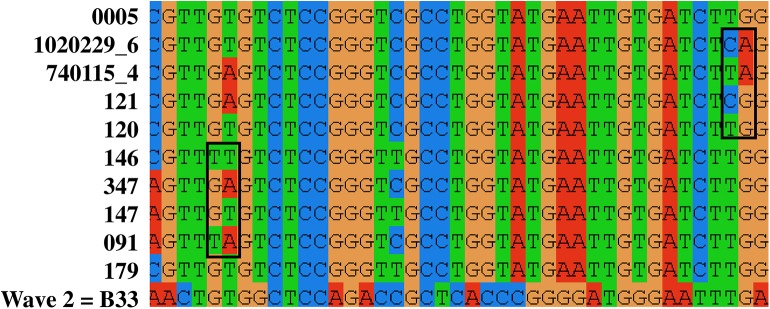
Diagram displaying only variable nucleotides among the 67 genomic sequences from Wave 3 of the 7^th^ pandemic from Manhiça and B33 (Genbank:ACHZ00000000), a Wave 2 isolate from Beira, Mozambique in 2004. Only variable nucleotides 80 to 120 are shown. Each row contains sequence from a single isolate. Two examples in which two loci with alleles occur in all four possible combinations are identified by boxes around the four chromosomes. These examples are consistent with recombination events.

Of the 71 isolates, the genomes of sixty-seven differed by less than 100 SNVs. Consistent with this, the core genome of all the wave 3 isolates shared 2802 genes. To look for evidence of recombination in these sequences we examined 2543 single copy conserved genes. We removed all of the invariant nucleotides, any SNVs in locally collinear blocks smaller than 200 bp, and examined the remaining variant sites in Clustal W (Fig 2). These data showed evidence of homoplasy by visual examination: for example there were multiple examples of dinucleotide sequences at the same site in different isolate genomes being present in all four possible combinations (e.g. AA, AG, GA, GG). This was despite the high level of pairwise nucleotide conservation between isolate genomes, usually differing by only 18 to 62 nucleotides. Among the 148 SNVs, 360 pairs were determined to have both alleles in all possible combinations. The probability of this occurring by mutation alone is vanishingly small (the probability of the same mutation on two different genomes to the 360^th^ power). The most parsimonious explanation for this observation is recombination within this population of *V*. *cholerae* O1s.

**Fig 3:**
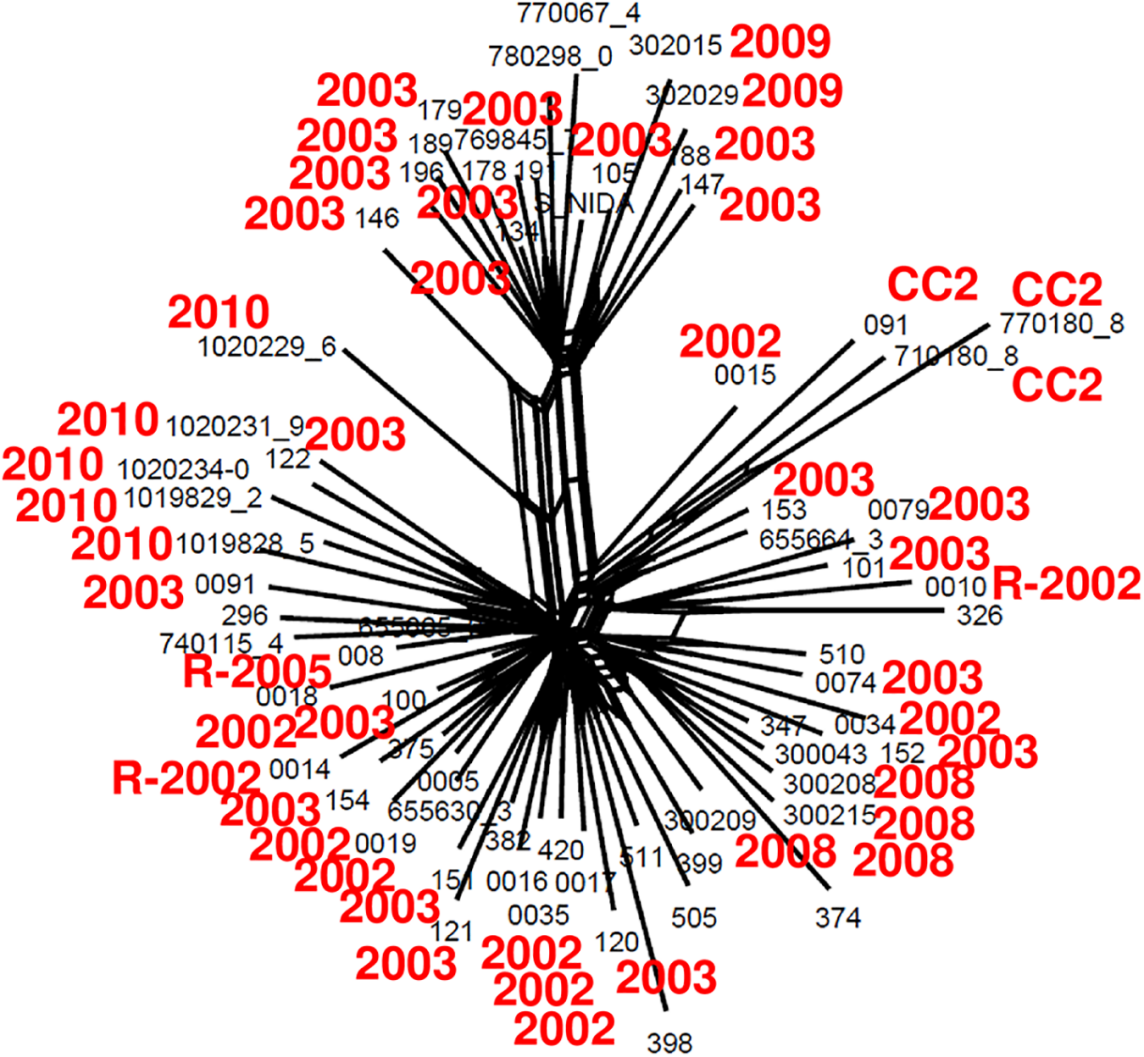
Splitstree network of wave 3 genome sequences from Manhiça, Mozambique. Splitstree allows for parallel changes whether they are derived by recurrent mutation or by recombination. The branch lengths are proportional to the number of nucleotide changes. The figure reveals extensive parallel changes (the parallelograms). The parallel lines of a parallelogram indicate potential recombination. Of note the isolates from 2008, those of 2009 and those of 2010 are in separate regions of the diagram and each has potential parallelograms in the diagrams. The lines extending to a single isolate represent the mutational variation. The smaller font indicates the isolate names. The four digit red numbers are the years in which different isolates were collected. The letter R identifies isolates found in the river. CC2 indicates those isolates found in MLVA CC2. The four isolates from 2008, the two isolates from 2009 and four of the five isolates from 2010 cluster with their contemporaries.

The genetic relatedness, including the recombinant loci, was estimated from WGS using a phylogenetic network [26]. As shown in Fig 3, while all of the genomes were distinct, there was some clustering by year of isolation, four of the five isolated in 2010 clustered together as did the four from 2008 and the two from 2009. The largest number of isolates in our collection were collected in 2003. It is evident from Fig 3 that they occupy positions throughout the network. This was also true for the three river isolates. In the network, every sequence had some nucleotide variants that distinguish it from every other sequence, thus in contrast to the MLVA network, there are no central genotypes that might be construed as the founder.

**Fig 4:**
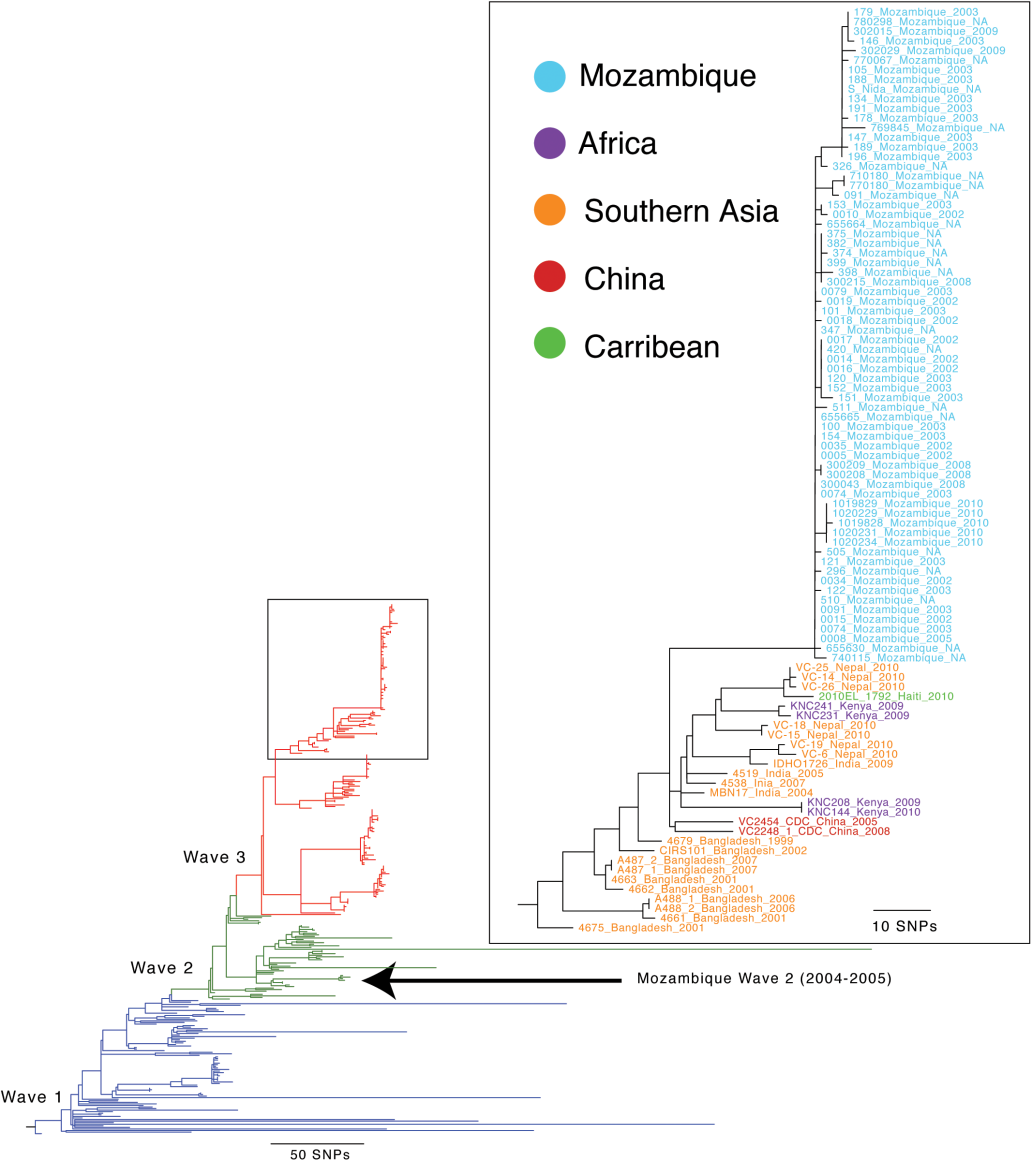
Phylogeny of 7^th^ pandemic isolates of *V*. *cholerae*. The branch lengths are proportional to the number of variable nucleotides. All of the isolates from Manhiça cluster together and are most closely related to isolates from India, Nepal, Kenya, Haiti and Bangladesh. Other isolates from Mozambique and Kenya are more distantly related.

Fig 4 shows the phylogenetic tree with genomic sequences from a global collection of 274 previously published sequences (S1 Table) including the 67 genomic sequences generated in this study. These data show that the sequences of the Manhiça, Mozambique isolates cluster in a monophyletic group distinct by 39 nucleotides from the backbone of the third wave of the Seventh Pandemic phylogeny (S2 Table). All Seventh Pandemic isolates harbor virulence associated genes, such as the CTX prophage, the genomic islands VSP-I and VSP-II, the toxin-coregulated pilus, the toxic linked cryptic element, and the integrative conjugative element SXT harboring multidrug resistance genes. The CTX phage harbours the *ctxB*^*cla*^ allele within an otherwise El Tor biotype CTX phage sequence and is typical of isolates referred to as “atypical” El Tor biotypes [32].

**Fig 5:**
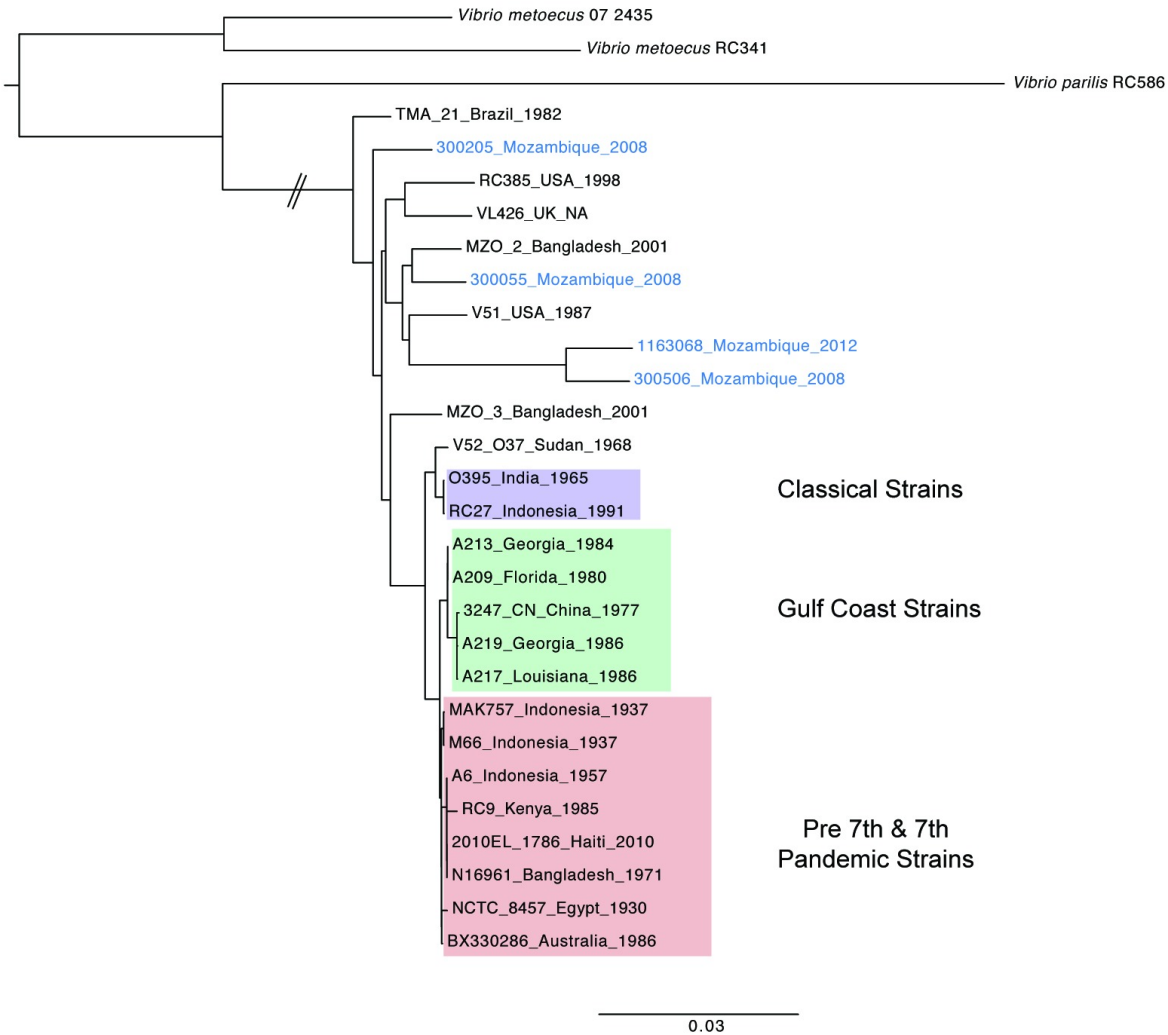
Phylogeny of the four isolates with singleton MLVA genotypes and >24,000 nucleotide differences compared to the 7^th^ pandemic isolates of *V*. *cholerae*. The branch lengths are proportional to the number of variable nucleotides.

The four sequences that differed by >24,000 nucleotides among the 3,237,973 basepairs conserved in all our sequenced genomes did not cluster with sequences from the Seventh Pandemic (Fig 5) and are quite distantly related to each other, but all were more closely related to the other *V*. *cholerae* sequences compared to other species. These isolates were taken from patients admitted to hospital with suspected cholera indicating that these divergent lineages are capable of causing clinical symptoms, as has been reported previously [13]. Unlike the Seventh Pandemic strains, these four strains do not contain any of the aforementioned virulence associated genes, although they contain a few genes, such as the RTX toxin gene cluster and the hemolysin *hlyA*, from some of the virulence associated islands.

The network based on WGS included isolates of both CCs (CC1 and CC2), however while the isolates of CC1 was distributed through the network, the isolates of CC2 clustered together (Fig 3). In the same way, the four singletons isolates by MLVA did not cluster in the network and were quite distantly related by WGS analysis.

## Discussion

Cholera remains important public health problem in Mozambique. We characterized *V*. *cholerae* isolates using MLVA and WGS to determine the genetic relatedness and transmission dynamics of cholera outbreaks in the Manhiça District. Our analyses of WGS data revealed that 94% of the isolates were a monophyletic group in the third wave of the Seventh Pandemic. These isolates have formed a locally evolving population that has persisted for at least eight years, either in a local environmental reservoir or circulating within the human population, and sporadically caused recurrent disease in southern Mozambique. It is yet to be shown if these isolates are representative of those circulating outside of the study site across Mozambique.

Previous studies have reported the role of environmental factors, such as seasonal fluctuations, that influence the dynamics of *V*. *cholerae* in environmental reservoirs [33,34]. Of note, all of our isolates were collected during the first half of the year, January through July, with a peak in May. However, our study does not allow establishing a clear relationship between environmental strains and those causing cholera outbreak. The presence of four isolates that are not part of the Seventh Pandemic (differing by ~ 0.75% of the genome sequence) demonstrates that a diverse set of *V*. *cholerae* not linked to the Seventh Pandemic are causing a background, low level of sporadic disease in southern Mozambique, despite the absence of many of the major virulence determinants. This has been seen elsewhere including in the Gulf coast of the USA particularly in the 1980’s and 1990’s [32].

We detected the presence of recombination in our WGS data. Most analyses of WGS data detect recombination as a large number of SNVs in short sequence of DNA. Our method of detection, the four-gamete test applied to haploid genomes, removes the constraint of the SNVs occurring in a small region by simply looking at dinucleotide pairs located at any distance from each other. Since mutations occur at random, a dinucleotide sequence, say GG, can mutate at either of two positions for example: AG and GA. In order to further mutate to form AA, one of the positions must mutate a second time. However, in this instance it also possible to replace the original sequence in a single recombination event. We found 360 pairs of nucleotide positions across all our genomes at which all four combinations of alleles in the dinucleotides were found. The probability of this occurring by mutation is extremely small (the rate of mutation at the same nucleotide to the 360^th^ power). Our sample is unusual because it was collected in small area and in a short time frame. The geographical and temporal proximity of isolates is a prerequisite for recombination. *V*. *cholerae* meets other necessary prerequisites like having an intact mechanism for uptake of DNA and integration into the chromosome [38]. The clearest examples of the effectiveness of recombination can be found in serotype switching, a process known to be accelerated by chitin [39]. The limited amount of variation in our sample is consistent with the recombinant events occurring within this population of *V*. *cholerae* O1s.

Most isolates (91%) were distributed among the 20 genotypes of CC1 and 41% had the founder genotype, supporting the hypothesis of a common ancestor which subsequently differentiates into additional genotypes. In a study of 187 isolates conducted in Haiti, only 9 MLVA genotypes clustered in a single clonal complex and 53% had the founder genotype [35].

We found more alleles (ten) at the first locus (VC0147) on large chromosome than at the two loci on small chromosome (VCA0171 and VCA0283) with seven alleles for each one. Our findings are in contrast to previous descriptions that show the three loci on the large chromosome varying at a slower rate than those on the small chromosome [11]. Extensive genetic variation on both chromosomes has been reported [36], but not more variation at the large chromosome loci [35–37].

WGS and MLVA patterns were performed to discriminate *V*. *cholerae* isolates from various geographic locations and distinct populations [17,40]. But, none of the 26 MLVA genotypes that we found in this study has been reported in previous studies from Haiti, Thailand, Bangladesh, India, Vietnam and Mozambique [10,11,35,36,40,41]. However, the three loci on large chromosome are likely to be considered the best for estimating across large distances [35]. Thus, when we considered the MLVA profiles in terms of the three loci (VC0147, VC0437, and VC1650) on large chromosome, the isolates with profiles 8,4,6, (50%, 39/78) and 9,4,6, (10%, 8/78) were related to the Haiti and Bangladesh strains, respectively [11,35]. Our WGS analysis demonstrates that these Mozambican isolates formed a distinct lineage within a clade that includes El Tor variants from Bangladesh, China, Haiti, Nepal, India, and Kenya that belong to the current global radiation of the Seventh Pandemic. The Manhiça, Mozambique isolates differ from the wave 3 backbone by 39 nucleotides. Furthermore, previous reports demonstrated Wave 2 strains in Mozambique during the same time period [21,42]. Taken together with our analysis, it is clear that there were multiple, independent *V*. *cholerae* lineages from Wave 2 and Wave 3 which were circulating within Mozambique during this period of time. In Manhiça, although the relationships between isolates differed in detail between MLVA and WGS, both analyses demonstrated the isolates were very closely genetically related, even if they are collected several years apart.

## Conclusion

Our findings further define the molecular epidemiology of *V*. *cholerae* in Mozambique. Our study demonstrates that Wave 3 isolates of the Seventh Pandemic have become established in Manhiça, Mozambique and have persisted in this region over the time of this study alongside *V*. *cholerae* Wave 2 strains in other regions of the country. The subsequent radiation of genotypes has been enriched by the process of recombination as detected in the WGS data. Our data raises several important questions that relate to where these *V*. *cholerae* isolates persist and how seemingly identical isolates can be collected years apart despite our understanding of the high rate of change of the MLVA loci and the *V*. *cholerae* molecular clock. Although the environmental triggers for the emergence of cholera are unknown in Manhiça, it is important to be vigilant to prevent an emergence from becoming an outbreak.

## Supporting information

S1 Table Isolates for 7th pandemic tree. (XLSX)

S2 Table List of 39 mutations that distinguish isolates from Manhiça, Mozambique from the Wave 3 backbone. (XLSX)
